# Mid-adolescent ethnic variations in overweight prevalence in the UK Millennium Cohort Study

**DOI:** 10.1093/eurpub/ckab023

**Published:** 2021-04-24

**Authors:** Michelle Stennett, Alex Blokland, Richard G Watt, Anja Heilmann

**Affiliations:** Department of Epidemiology and Public Health, University College London, London, UK

## Abstract

**Background:**

There are stark ethnic inequalities in the prevalence of UK childhood obesity. However, data on adolescent overweight in different ethnic groups are limited. This study assessed ethnic inequalities in overweight prevalence during mid-adolescence using body mass index (BMI) and explored the contribution of socioeconomic and behavioural factors.

**Methods:**

We analyzed data from 10 500 adolescents aged between 13 and 15 years who participated in sweep six of the Millennium Cohort Study. Ethnic inequalities in overweight and mean BMI were assessed using multiple regression models. Results were stratified by sex and adjusted for socioeconomic and behavioural factors.

**Results:**

Black Caribbean males had significantly higher BMI than White males after full adjustment [excess BMI 2.94, 95% confidence interval (CI) 0.70–5.19] and were over three times more likely to be overweight [odds ratio (OR): 3.32, 95% CI 1.95–5.66]. Black Africans females had significantly higher BMI compared with White females (excess BMI 1.86, 95% CI 0.89–2.83; OR for overweight 2.74, 95% CI 1.64–4.56), while Indian females had significantly lower BMI compared with White females (reduced BMI −0.73, 95% CI −1.37 to −0.09). Socioeconomic and behavioural factors often considered to be associated with overweight were more prevalent in some ethnic minority groups (lower socioeconomic position, lack of breakfast consumption, low fruit and vegetable intake, high sugar-sweetened beverage and fast-food consumption, and infrequent physical activity), but adjustment for these factors did not fully explain ethnic differences in overweight/BMI.

**Conclusion:**

Ethnic inequalities in overweight prevalence are evident in mid-adolescence and vary according to sex. Differences in overweight/BMI between ethnic groups were not fully accounted for by socioeconomic or behavioural factors.

## Introduction

The dramatic global rise in the prevalence of childhood obesity over the past 30 years has been described as ‘one of the most serious public health challenges of the 21st century’.^1^ The UK has one of the highest prevalence rates of adult overweight and obesity in Europe^2^; furthermore, substantial rises in prevalence are predicted over the next 30 years.^3^ Obese children are five times more likely to become obese adults,^4^ and there are known associations between adult obesity and various chronic diseases.^3^ This has important implications for healthcare provision and the associated increased economic burdens on the National Health Service.^5^ In addition to these long-term concerns, there are well-documented adverse health consequences of obesity in childhood itself; such as increased risk of hypertension, asthma and psychological problems.^6^ Research into childhood overweight and obesity not only focuses on management but also explores potential explanatory factors; including parental socioeconomic position (SEP),^7^ low birthweight^8^ and behavioural factors such as decreased physical activity and dietary composition.^9^

Current evidence shows increased overweight rates in some UK ethnic minority groups; with higher prevalence of excess weight often found in Black children compared with White children.^9–11^ Findings for children from South Asian groups appear to be inconsistent, with some studies demonstrating higher overweight and obesity prevalence,^9^^,^^12^ and others showing similar or lower prevalence when compared with White children.^13^^,^^14^

Research has also shown variations in overweight prevalence according to sex, often with higher prevalence in females.^7^^,^^9^ However, explanations for biological differences suggested in previous literature (possible higher levels of appetite suppressing hormones in females),^15^ and behavioural factors related to gender (lower calorie consumption in females due to weight concerns)^15^^,^^16^ do not seem to explain the higher prevalence of overweight seen in females across most ethnic groups. Many of these studies focus on younger children, and may indicate other unexplored factors related to sex and ethnicity, particularly in older age groups.

Adolescence is an important period where children begin to develop independence over food selection, whilst also undergoing changes in body structure that affect body fat.^17^ While 55% of pre-pubescent obese children remain obese into adolescence, 80% of obese adolescents remain obese into adulthood; making adolescence an important period to target public health obesity reduction strategies.^4^ There are relatively few studies on overweight and obesity in UK ethnic minorities during adolescence; many existing studies are based on regional data in England^7^^,^^9^^,^^13^ and some combine disparate ethnic groups into general classifications.^7^ Broad categorizations, such as ‘South Asian’ or ‘Black’, may mask important variations in overweight between sub-groups.^18^ In addition, there has been little exploration into the role of both socioeconomic and behavioural factors in UK ethnic minority adolescent obesity; with only one previous London-based study examining the contribution of behavioural ‘risk factors’ within this age group.^9^

This study seeks to address these limitations by utilizing a large, UK national dataset with ethnic minority representation in disaggregated groups. We explore ethnic differences in mid-adolescence overweight as measured by BMI, whilst also assessing the contribution of selected socioeconomic and behavioural factors.

## Methods

The Millennium Cohort Study (MCS) is a UK representative, longitudinal study. The initial sample included ∼19 000 UK born children between 2000 and 2002, and data have been collected from participants at 7 points in time (ages 9 months, 3, 5, 7, 11, 14 and 17 years). Cross-sectional interview data from the MCS at sweep 6 in 2015 (MCS6) were used in this study, with the addition of birthweight data from earlier sweeps in 2001 (MCS1), and 2004 (MCS2). The majority of cohort members was aged 14 years at the time of the outcome data collection. Supplementary figure S1 shows the sample sizes for each survey sweep from ages 3 to 14 years (MCS2 to MCS6). Reduced sample sizes across the survey sweeps have been attributed to several reasons including untraced moves, emigration and refusals. Non-response survey weights were constructed for MCS6 to account for survey attrition.^19^

The study employed a two-stage stratified clustered random sampling strategy, with oversampling in disadvantaged wards and in areas with more than 30% ‘Asian’ or ‘Black’ residents. Data on cohort members and parents are collected at each sweep via interviews and self-completion questionnaires. Ethical approval for MCS6 was granted from the National Research Ethics Committee London (REC 13/LO/1786).^20^

### Body mass index

Anthropometric data from cohort members were collected by trained interviewers. Prior to measurement, participants removed shoes, socks, outdoor clothing and hairstyles/accessories that could affect accuracy. Height was recorded to the nearest millimetre using a Leicester height measure stadiometer with the measurement arm parallel to the Frankfurt plane.^21^ Weight was recorded using Tanita™ scales (BF-522W) to the ∼0.1 kg. Measurements were only repeated if the interviewer was not happy with the initial measurement. Any possible factors that could have affected measurement accuracy were recorded by the interviewer.^21^

BMI was derived from height and weight measurements (BMI = weight kg height m^−2^) and used as a continuous variable. We also used the MCS derived overweight variable that is based on International Obesity Task Force (IOTF) BMI age and sex specific cut-off points.^22^ Overweight and obesity categories were combined into a single ‘overweight’ category within a binary variable (‘healthy weight’—including underweight vs. ‘overweight’—including obese).

### Ethnicity

Ethnic classification was derived from the cohort member questionnaire, where participants self-identified their ethnicity from predefined categories. We used eight categories in this analysis: White, Mixed, Indian, Pakistani, Bangladeshi, Black Caribbean, Black African and Other Ethnic group (comprising Chinese, Other Black, Other Asian and Other Ethnic group). White ethnicity was the reference category in regression analyses.

### Covariates

Covariates were selected *a priori* based on previous evidence for associations with both overweight and ethnicity. SEP was measured via parental education and family income. Parental education represents the highest qualification obtained by main parental respondents over all MCS sweeps. 94% of respondents were mothers; consequently, this variable reflects maternal education. The variable was categorized into six categories: No formal qualifications, overseas qualification, GCSE below grade C (NVQ 1), GCSE grade A-C (NVQ 2), A/AS Level (NVQ 3) and Postgraduate degree or degree (NVQ 4/5). Family income was categorized into equivalized income quintiles, ordered from highest earning to lowest. Birth weight in kilograms was used as a continuous variable for all regression analyses. For descriptive purposes, birth weight was grouped into three categories (high, normal and low) based on Office for National Statistics classifications.^23^ Dietary and behavioural factors [consumption of breakfast, fruit, vegetables, sugar-sweetened beverages (SSB), fast-food, and physical activity] were self-reported by cohort members and were all categorical variables (see Supplementary Appendix S1 for categories). A combined category of ‘Less than once a month, hardly ever or never’ was used for SSB due to small numbers in some categories. Physical activity was recategorized as ‘5 days or more’, ‘3–4 days’, ‘1–2 days’ or ‘Not at all’ for similar reasons.

### Data analyses

All analyses were performed using STATA version 15^24^ and MCS6 UK wide cross sectional survey weights to account for sample attrition across sweeps and the stratification and geographical clustering of the sample. Statistical significance was predetermined as *P* = 0.05.

The initial sample included 11 884 participants (11 726 families). Twins and triplets were excluded (*n* = 158) due to the associations between multiple births and low birth weight and possible impact on childhood overweight.^25^ Nine observations with missing data for the income variable also had missing values for the survey characteristics and were removed. Finally, 1217 observations were removed due to missing data for any covariate for complete case analysis, giving a final sample size of 10 500 (tables 1–3).

**Table 1:** Descriptive statistics of ethnicity by covariates (*N* = 10 500)

	Ethnic group							
	White (*n* = 8398)	Mixed (*n* = 485)	Indian (*n* = 284)	Pakistani (*n* = 512)	Bangladeshi (*n* = 231)	Black Caribbean (*n* = 100)	Black African (*n* = 199)	Other (*n* = 291)
BMI, mean (SE)	21.46 (0.06)	21.49 (0.30)	20.84 (0.26)	21.88 (0.25)	21.88 (0.33)	23.60 (0.74)	21.95 (0.56)	21.70 (0.33)
Overweight (%)	26.3	24.7	21.5	34.7	32.2	44.3	37.9	29.4
Sex (%)								
Male	51.8	57.0	57.3	49.7	50.1	64.9	53.4	49.2
Female	48.2	43.0	42.7	50.3	49.9	35.1	46.6	50.8
Age (%)								
13	22.9	30.3	28.1	21.4	30.8	19.2	24.3	26.6
14	75.8	68.7	67.5	77.6	67.3	80.8	74.5	70.7
15	1.3	1.0	4.4	1.0	1.9	–	1.2	2.7
Maternal education (%)								
NVQ 4/5	39.7	46.9	38.8	17.6	13.9	40.7	36.3	23.2
NVQ 3	14.8	12.0	14.3	11.2	10.4	15.0	10.7	9.9
NVQ 2	27.8	22.9	16.7	18.0	16.0	23.4	12.3	18.0
NVQ 1	7.3	4.5	2.9	7.4	5.9	3.8	6.1	3.2
Overseas	2.0	3.2	7.7	12.1	20.5	1.8	8.6	12.5
None of these	8.5	10.5	19.6	33.7	33.1	15.4	26.1	33.2
Income quintile (%)								
Highest	23.9	20.4	20.4	0.1	2.2	3.5	6.6	9.8
Fourth	22.9	17.2	24.8	1.7	2.2	16.6	9.5	14.3
Third	21.3	20.3	20.9	10.2	4.8	20.1	14.0	21.3
Second	18.6	20.5	23.0	22.1	19.4	17.3	21.8	20.3
Lowest	13.4	21.6	10.9	65.8	71.3	42.5	48.2	34.3
Birth weight (%)								
Low (<2.5 kg)	6.7	7.5	14.5	10.8	10.7	12.7	10.4	5.7
Normal (≥2.5–4 kg)	82.4	81.5	81.1	82.4	85.5	81.3	81.8	90.4
High (>4 kg)	10.9	11.0	4.3	6.8	3.8	6.0	7.8	3.9
Breakfast consumption (over a week) (%)								
Every day	51.8	45.8	72.2	60.9	50.1	34.8	49.6	49.8
Some days	38.3	43.4	24.0	32.1	46.0	55.0	42.5	42.0
Never	9.9	10.8	3.8	7.0	3.8	10.2	7.9	8.2
Fruit consumption (at least 2 portions) (%)								
Every day	29.5	26.0	40.5	29.1	24.1	5.9	18.6	29.5
Some days	60.8	64.5	55.3	64.8	68.8	83.4	74.1	67.0
Never	9.6	9.4	4.2	6.1	7.1	10.7	7.3	3.5
Vegetable consumption (at least 2 portions) (%)								
Every day	38.2	38.2	37.4	18.8	21.7	26.7	35.4	35.8
Some days	53.5	55.7	57.9	73.1	69.2	65.7	55.6	57.1
Never	8.3	6.1	4.7	8.1	9.2	7.6	9.0	7.1
Sugar-sweetened beverage consumption (%)								
Once a day or more	25.3	26.3	12.8	33.8	28.7	25.3	20.3	22.8
3–6 days a week	21.1	17.4	27.0	22.4	17.0	24.6	20.4	22.1
1–2 days a week	24.0	23.4	27.4	25.8	31.1	22.9	32.6	25.4
Once a month	12.9	11.7	13.4	10.2	14.1	16.1	14.4	12.1
<Once a month	16.7	21.2	19.4	7.8	9.1	11.1	12.3	17.6
Fast food consumption (%)								
Once a day or more	1.5	5.0	1.1	3.7	2.7	1.9	2.8	4.0
3–6 days a week	3.5	4.9	6.6	10.8	7.7	16.4	11.7	8.4
1–2 days a week	21.0	26.8	22.2	41.1	41.5	40.3	25.0	27.6
Once a month	45.9	40.9	45.8	30.8	38.1	27.0	36.9	36.0
<Once a month	23.8	20.2	21.4	10.9	5.8	11.1	21.3	19.7
Hardly ever/never	4.3	2.2	2.8	2.7	4.1	3.1	2.3	4.3
Physical activity (at least 1 h of moderate activity over the past week) (%)								
5 days or more	37.8	43.8	37.9	34.4	36.6	26.1	38.1	44.8
3–4 days	34.0	29.4	33.8	31.5	31.5	35.6	29.4	29.4
1–2 days	23.5	21.1	24.6	30.3	29.2	30.9	23.0	23.0
Not at all	4.7	5.7	3.7	3.8	2.6	7.5	9.5	2.9

All figures are proportions unless otherwise stated. Proportions and means weighted.

BMI, body mass index; NVQ, National Vocational Qualification.

**Table 2:** Multiple logistic regression of overweight^a^ stratified by sex (*N* = 10 500)

Ethnic group^b^	Model A	Model B	Model C	Model D
Males (*n* = 5290)				
Mixed	0.92 (0.64–1.33)	0.89 (0.61–1.28)	0.95 (0.65–1.39)	0.96 (0.65–1.40)
Indian	1.00 (0.61–1.63)	0.98 (0.59–1.65)	1.11 (0.69–1.80)	1.25 (0.76–2.06)
Pakistani	1.48 (1.08–2.02)	1.13 (0.79–1.61)	1.18 (0.83–1.68)	1.30 (0.91–1.85)
Bangladeshi	1.21 (0.74–2.00)	0.92 (0.53–1.60)	0.96 (0.57–1.62)	1.00 (0.58–1.73)
Black Caribbean	3.45 (1.81–6.58)	2.93 (1.59–5.42)	2.88 (1.65–5.04)	3.32 (1.95–5.66)
Black African	1.07 (0.68–1.68)	0.90 (0.54–1.52)	0.98 (0.58–1.64)	1.01 (0.59–1.72)
Other ethnic group	1.31 (0.82–2.10)	1.14 (0.70–1.87)	1.25 (0.77–2.04)	1.25 (0.77–2.03)
Females (*n* = 5210)				
Mixed	0.94 (0.64–1.39)	0.91 (0.61–1.36)	0.93 (0.62–1.40)	0.99 (0.65–1.49)
Indian	0.54 (0.29–1.02)	0.55 (0.29–1.07)	0.57 (0.30–1.11)	0.60 (0.30–1.18)
Pakistani	1.50 (1.11–2.04)	1.21 (0.88–1.69)	1.22 (0.87–1.72)	1.41 (0.98–2.02)
Bangladeshi	1.45 (0.89–2.34)	1.19 (0.71–1.98)	1.23 (0.73–2.06)	1.39 (0.79–2.46)
Black Caribbean	1.00 (0.50–1.97)	0.90 (0.44–1.82)	0.90 (0.45–1.83)	1.02 (0.52–1.98)
Black African	2.76 (1.67–4.56)	2.49 (1.48–4.19)	2.50 (1.46–4.27)	2.74 (1.64–4.56)
Other ethnic group	1.06 (0.66–1.69)	0.99 (0.61–1.61)	1.01 (0.61–1.65)	1.01 (0.60–1.69)

Values displayed are odds ratios (95% CI).Model A: crude association between ethnicity and BMI, age. Model B: Model A, additional adjustment for maternal education, family income.

Model C: Model B, additional adjustment for birth weight, physical activity. Model D: fully adjusted-Model C, additional adjustment for dietary variables.

a Overweight includes obese participants. Healthy weight (including underweight) is the reference group.

b White ethnicity is the reference group.

**Table 3:** Multiple linear regression of body mass index (BMI) stratified by sex (*N* = 10 500)

Ethnic group^a^	Model A	Model B	Model C	Model D
Males (*n* = 5290)				
Mixed	−0.07 (−0.63 to 0.50)	−0.15 (−0.72 to 0.42)	−0.02 (−0.57 to 0.53)	−0.05 (−0.63,0.52)
Indian	−0.11 (−0.89 to 0.68)	−0.12 (−0.91 to 0.67)	0.10 (−0.61 to 0.82)	0.36 (−0.36,1.09)
Pakistani	0.39 (−0.40 to 1.18)	−0.17 (−1.03 to 0.69)	−0.09 (−0.92 to 0.73)	0.14 (−0.68,0.96)
Bangladeshi	0.40 (−0.65 to 1.45)	−0.21 (−1.31 to 0.89)	−0.14 (−1.20 to 0.92)	−0.02 (−1.07,1.04)
Black Caribbean	3.17 (0.77 to 5.56)	2.80 (0.45 to 5.15)	2.74 (0.46 to 5.02)	2.94 (0.70,5.19)
Black African	−0.73 (−1.87 to 0.41)	−1.06 (−2.26 to 0.14)	−0.91 (−2.08 to 0.27)	−0.76 (−1.84,0.32)
Other ethnic group	0.22 (−0.68 to 1.12)	−0.04 (−0.97 to 0.89)	0.10 (−0.80 to 1.00)	0.16 (−0.71,1.03)
Females (*n* = 5210)				
Mixed	0.35 (−0.71 to 1.42)	0.29 (−0.78 to 1.36)	0.35 (−0.72 to 1.42)	0.43 (−0.63,1.49)
Indian	−1.16 (−1.74 to −0.59)	−1.06 (−1.66 to −0.45)	−0.88 (−1.50 to −0.26)	−0.73 (−1.37,−0.09)
Pakistani	0.40 (−0.44 to 1.24)	−0.06 (−0.94 to 0.81)	−0.02 (−0.91 to 0.88)	0.31 (−0.64,1.26)
Bangladeshi	0.46 (−0.48 to 1.39)	0.07 (−0.90 to 1.04)	0.20 (−0.78 to 1.19)	0.45 (−0.60,1.50)
Black Caribbean	0.57 (−0.51 to 1.64)	0.29 (−0.78 to 1.36)	0.40 (−0.71 to 1.51)	0.66 (−0.40,1.71)
Black African	1.92 (0.94 to 2.89)	1.67 (0.65 to 2.68)	1.66 (0.62 to 2.69)	1.86 (0.89,2.83)
Other ethnic group	0.24 (−0.72 to 1.20)	0.18 (−0.78 to 1.15)	0.25 (−0.73 to 1.22)	0.36 (−0.62,1.34)

Values displayed are coefficient estimates (95% CI).

Model A: crude association between ethnicity and BMI, age. Model B: Model A, additional adjustment for maternal education, family income.

Model C: Model B, additional adjustment for birth weight, physical activity. Model D: fully adjusted-Model C, additional adjustment for dietary variables.

a White ethnicity is the reference group.

Comparisons were made between characteristics of participants with any missing data and those with complete data (Supplementary table S1). In total, participants with missing data amounted to just over 10% of the original sample size. There were no significant differences between both groups in terms of the outcome variables, birth weight or sex. However, the missing data group had a significantly higher proportion of ethnic minorities, older participants and those from disadvantaged households (belonging to the lowest income quintile, mother having no educational qualifications). Those with missing data were also more likely to never consume breakfast or vegetables and to never participate in at least one hour of moderate physical activity within a week.

Multiple linear regression was used to model the relationships between ethnicity and BMI. Logistic regression models were generated for the binary overweight variable as the outcome and ethnicity as the explanatory variable. Regression results were stratified by sex due to a significant interaction term for the BMI outcome (F = 3.28, *P* = 0.002). For both outcomes, a series of regression models was estimated. Model A was the baseline model, estimating the association between ethnicity and overweight/BMI, adjusted for age. Model B additionally adjusted for SEP markers (maternal education and family income), Model C additionally adjusted for birth weight and physical activity. Model D fully adjusted for all variables including dietary variables.

## Results

Table 1 shows descriptive statistics, with weighted proportions and means. 2102 cohort members belonged to an ethnic minority group (20% of the total sample), of which Pakistani participants formed the majority.

All ethnic minority groups had higher proportions of overweight adolescents than the White group, apart from the Mixed and Indian groups. Black Caribbean adolescents had the highest proportion of overweight adolescents (44.3%), mean BMI (23.60). The relationship between ethnicity and overweight differed according to sex, most notably in ethnic minority groups (Supplementary figures S2 and S3). Black Caribbean males had an overweight prevalence of 53.3%, whilst Black Caribbean females had a lower prevalence of 27.7%. In contrast, Black African females had a prevalence of 51.5%, whilst Black African males had a lower prevalence of 26.2%. Indian males had a higher overweight prevalence (24.6%) than Indian females (17.4%).

There were also variations in the proportions of ethnic minority participants within the covariates (table 1). Larger proportions of ethnic minority groups were represented within the lowest income quintile, containing more than two thirds of Pakistani and Bangladeshi adolescents. In comparison to White adolescents, low birth weight was more prevalent among most ethnic minority groups. Dietary and physical activity behaviours considered to be associated with overweight (lack of breakfast consumption, low fruit and vegetable intake, high SSB and fast-food consumption, low physical activity) were all more prevalent in ethnic minority groups.

Table 2 displays logistic regression models estimating the odds of being overweight. For males, unadjusted odds of overweight were significantly higher for both Pakistani (OR 1.48, 95% CI 1.08–2.02) and Black Caribbean adolescents (OR 3.45, 95% CI 1.81–6.58) in comparison to White adolescents. When adjusted for SEP, Pakistani males no longer had significantly higher odds of overweight in comparison to White males. Adjusting for SEP, birthweight and physical activity reduced the odds of overweight for Black Caribbean males, although the difference in comparison to White males was not fully explained even after full adjustment (Model D). In females, both Pakistani (OR 1.50, 95% CI 1.11–2.04) and Black African adolescents (OR 2.76, 95% CI 1.67–4.56) had significantly higher unadjusted odds of overweight in comparison to White adolescents (Model A). After accounting for SEP, the difference between Pakistani and White females was no longer statistically significant, but Black African females continued to display significantly higher odds of overweight in all models.

Table 3 displays regression models predicting BMI. In the baseline model (Model A), Black Caribbean males had a significantly higher BMI (excess BMI 3.17, 95% CI 0.77–5.56) compared with White males. This difference remained statistically significant in all models. Although the estimate was slightly attenuated following adjustment for family SEP markers, the addition of birth weight and physical activity had little effect. Black African females had a significantly higher BMI than White females (excess BMI 1.92, 95% CI 0.94–2.89), whilst Indian females had a significantly lower BMI than White females (reduced BMI −1.16, 95% CI −1.74 to −0.59) before adjustments (Model A). Accounting for family SEP markers resulted in attenuation of both estimates, which remained significant. Adding birth weight, physical activity and dietary variables to the model further attenuated the association among Indian females, but not among Black African females.

## Discussion

Our findings show inequalities in BMI and overweight prevalence during mid-adolescence between UK ethnic groups. Within this sample, ethnic differences in overweight patterns varied according to sex. In comparison with their White counterparts, Black Caribbean males had higher odds of overweight, whilst Black African females had higher odds of overweight. Indian females were the only ethnic group to have significantly lower BMI than White females. Ethnic differences in childhood overweight between males and females have previously been identified in research, with higher prevalence often reported in Black females, as seen in these data.^7^^,^^9^^,^^26^

Reporting BMI prevalence in disaggregated ethnic groups has revealed some distinctions, such as the contrast in levels of overweight between Black Caribbean and Black African adolescents and between Indian and Pakistani/Bangladeshi adolescents. These ethnic groups are often analyzed together as in some previous adolescent studies,^7^ thus masking such inequalities. A similar, but slightly smaller study also assessed overweight prevalence in disaggregated ethnic groups and found both Black Caribbean and Black African females had higher prevalence of overweight in comparison to White females.^9^ However, this study^9^ also found that other males had higher prevalence of overweight in comparison to White males, which contrasts with our findings. Such distinctions may be relevant information for targeted obesity interventions, but larger scale studies with higher representation of these disaggregated adolescent ethnic groups are needed to verify the findings of this research.

There were clear, stepwise social gradients in BMI by family income for both sexes, but no similar gradients were observed by maternal education. SEP explained part of the association between ethnicity and BMI, except for Indian males. It appears that the high prevalence of deprivation in Pakistani and Bangladeshi groups (see table 1) is a major factor for these inequalities in BMI prevalence.

In the previous research, the role of SEP in explaining ethnic differences in overweight and obesity has been inconsistent. Some previous smaller scale studies involving adolescent ethnic groups in England did not find significant associations between SEP and BMI in samples including adolescent ethnic groups.^26^^,^^27^ However, in line with our findings, a previous MCS study in younger children also found SEP adjustments attenuated increased overweight prevalence in 5-year-old Bangladeshi children to non-significant levels.^28^ In the current study, SEP adjustments did not ultimately explain all ethnic group variation in overweight and BMI, with higher levels remaining particularly among Black compared with White groups. Furthermore, recent research has found that the association between SEP and overweight is reversed in some UK ethnic groups, with children from lower income Black African and Black Caribbean groups having a lower risk of overweight than higher income families from the same ethnic group.^29^

Although high birth weight, frequent physical activity and frequent breakfast consumption were associated with lower BMI, these factors did not play a substantial role in explaining differences in BMI between ethnic groups. A previous study examining the role of dietary factors in adolescent obesity found poor dietary behaviours, including skipping breakfast, more prevalent in Black African females, alongside high levels of overweight.^9^ In this study, unhealthy behaviours (skipping breakfast, infrequent fruit and vegetable consumption, frequent SSB and fast-food consumption and infrequent physical activity) did not explain the patterns of obesity prevalence observed.

Vegetable consumption was not associated with BMI in any model, and the frequent consumption of SSB was only associated with higher odds of overweight in males. Contrary to expectations, both lower fruit consumption and higher fast-food consumption were associated with lower BMI. This may be due to reporting bias, with the possibility that adolescents who were overweight reported less frequent fast-food consumption and higher fruit consumption, in line with well-known general health messages regarding obesity. The reverse association in fast-food consumption has been reported previously in international studies on adolescents.^30^

### Limitations

Although oversampled, there were low numbers for some ethnic minority groups within the sample, with implications for statistical power. The findings regarding higher levels of overweight/BMI in Black Caribbean males and Black African females are statistically significant, but the associated 95% confidence intervals are large due to small numbers in each group (tables 2 and 3 and Supplementary figures S2 and S3). Additionally, a growing body of evidence highlights the limitations of BMI to account for variant body compositions in children from different ethnic groups.^31^^,^^32^ BMI does not account for lean body mass and may have the propensity to underestimate body fat in South Asian groups and overestimate body fat in Black groups.^31^ This may have implications for the validity of the estimated overweight levels in these analyses. Unfortunately, there are no current validated ethnic specific adjustments for children over the age of 12 years that could be applied to our data.

The crude nature of the dietary questions included in MCS (Supplementary Appendix S1) did not allow for an assessment of total energy consumption and may not accurately reflect dietary patterns. It may well be easier to recall if breakfast was consumed or not, than the exact frequency of fruit and vegetable consumption over a day. Furthermore, portion sizes were not accounted for in the SSB or fast-food questions, and it is not clear whether other dietary practices not measured by these variables (such as macronutrient intake of fats and sugar or frequency of snacking) may play a more significant role in explaining overweight/BMI differences. Consequently, these findings should be interpreted with caution.

### Research and policy implications

The most recent census data from England and Wales revealed that 14% of the population belonged to an ethnic minority group^33^; this is projected to increase to 25% of the population by 2051.^34^ In light of the anticipated UK ethnic population expansion and increased childhood excess weight prevalence within some ethnic groups, it is important to identify high-risk groups accurately to inform obesity reduction strategies. Moreover, further research is needed to examine other possible contributory factors not examined here, such as cultural or gender differences in body image.^16^ These may help to explain the higher prevalence of overweight in South Asian and Black ethnic adolescent groups, particularly Black African females who consistently show higher prevalence in research.

## Conclusions

This research adds to the limited evidence base regarding overweight prevalence within UK ethnic groups during mid-adolescence. It highlights further the complexities around identifying significant contributory factors to excess weight prevalence in minority groups. Further detailed research is required to investigate possible explanatory factors for higher overweight prevalence in ethnic minority adolescents in order for effective intervention strategies to tackle adolescent overweight.

## Supplementary Material

ckab023_Supplementary_Data
