# Supplementary material for: Mid-adolescent ethnic variations in overweight prevalence in the UK Millennium Cohort Study
Source: Eur J Public Health. 2021 Apr 24;31(2):396–402. doi: 10.1093/eurpub/ckab023 (PMC8565477; doi:10.1093/eurpub/ckab023)
Supplement: ckab023_Supplementary_Data [file ckab023_supplementary_data.zip › ejph-2020-03-om-0274-File002.docx]

**Supplementary Figure 1**. MCS Sample size by sweep

MCS 2 MCS3 MCS4 MCS5 MCS6

Age 3 Age 5 Age 7 Age 11 Age 14

Number of participant families


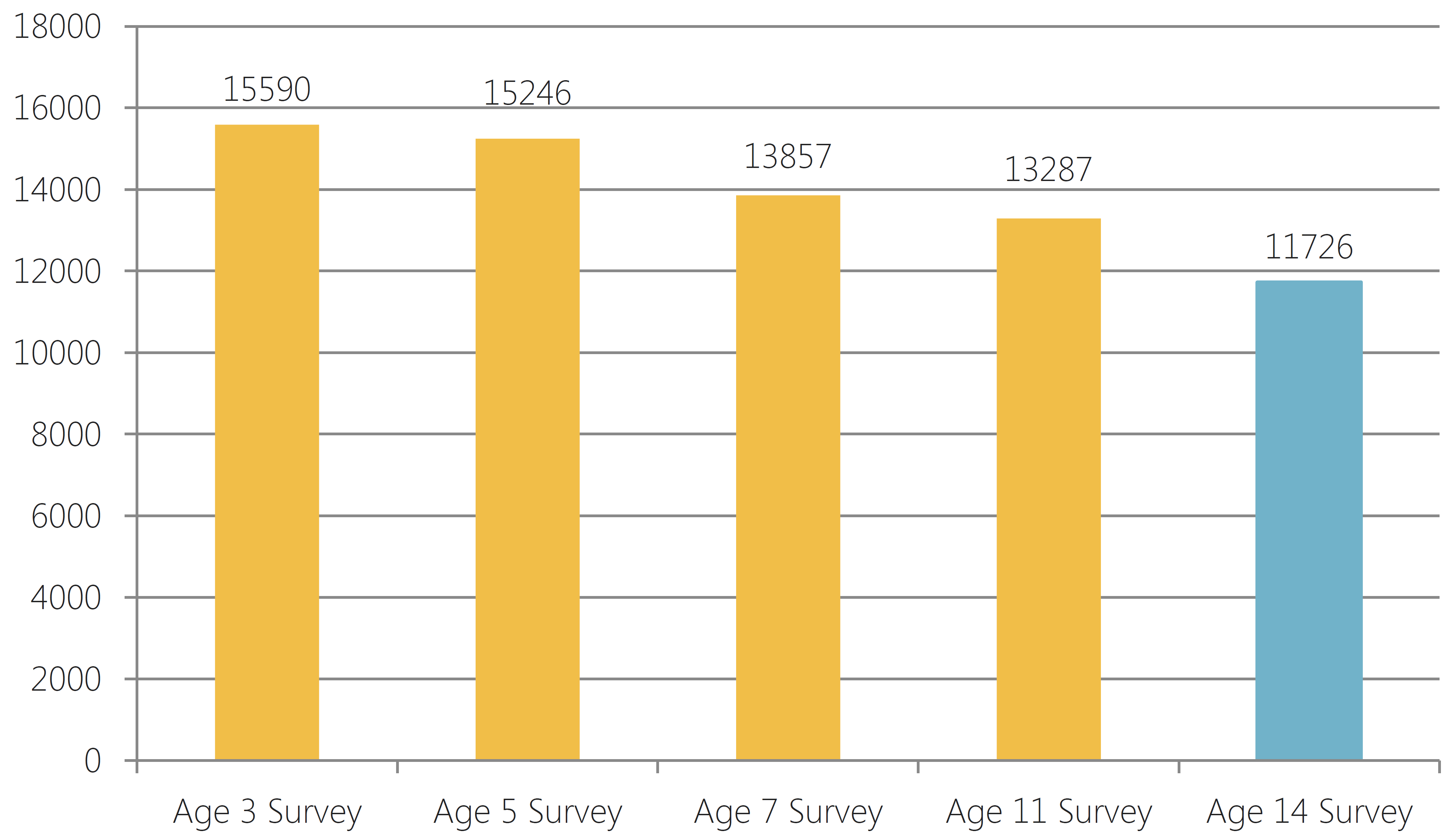


***Source:*** Burston K, Cleary A, Colin G, Michelmore O, Sheriffs P, Conolly A, et al. Millennium Cohort Study Sixth Sweep (MCS6). Technical Report (version 2) 2017. Ipsos MORI Social Research Institute. Available at: <http://doc.ukdataservice.ac.uk/doc/8156/mrdoc/pdf/mcs6_technical_report.pdf>.
