# Supplementary material for: Mid-adolescent ethnic variations in overweight prevalence in the UK Millennium Cohort Study
Source: Eur J Public Health. 2021 Apr 24;31(2):396–402. doi: 10.1093/eurpub/ckab023 (PMC8565477; doi:10.1093/eurpub/ckab023)
Supplement: ckab023_Supplementary_Data [file ckab023_supplementary_data.zip › ejph-2020-03-om-0274-File003.docx]

**Supplementary Appendix 1**.

**Millennium Cohort Study sweep 6: Young Person Questionnaire Health behaviour questions**

***Breakfast consumption***

*How often do you eat breakfast over a week?*

1. Never
2. Some days, but not all days
3. Every day

***Fruit consumption***

*How often do you eat at least 2 portions of fruit per day? A portion of fruit could be a whole piece of fruit, like an apple or banana or 80g of fruit (like in a fruit salad) but* ***does not include fruit juices***.

1. Never
2. Some days, but not all days
3. Every day

***Vegetable consumption***

*How often do you eat at least 2 portions of vegetables including salad, fresh, frozen or tinned vegetables per day?*

A portion is 3 heaped tablespoons of cooked vegetables or beans /pulses or a handful of cherry tomatoes or a small bowl of salad. **It does not include potatoes**.

1. Never
2. Some days, but not all days
3. Every day

***Sugar sweetened beverage consumption***

*How often, if at all, do you drink sugary drinks like regular cola or squash?*

1. More than once a day
2. Once a day
3. 3-6 days a week
4. 1-2 days a week
5. Less often but at least once a month
6. Less than once a month
7. Hardly ever or never

**Supplementary Appendix 2 continued…**

***Fast food consumption***

*How often, if at all, do you eat fast food such as McDonalds, Burger King, KFC or other fast food like that?*

1. More than once a day
2. Once a day
3. 3-6 days a week
4. 1-2 days a week
5. Less often but at least once a month
6. Less than once a month
7. Never

***Physical Activity***

*On how many days in the last week did you do a total of* ***at least an hour*** *of moderate to vigorous physical activity? By* ***moderate to vigorous*** *we mean any physical activity that makes you get warmer, breathe harder and makes your heart beat faster, e.g. riding a bike, running, playing football, swimming, dancing, etc.*

1. Every day
2. 5-6 days
3. 3-4 days
4. 1-2 days
5. Not at all
