# Supplementary material for: Mid-adolescent ethnic variations in overweight prevalence in the UK Millennium Cohort Study
Source: Eur J Public Health. 2021 Apr 24;31(2):396–402. doi: 10.1093/eurpub/ckab023 (PMC8565477; doi:10.1093/eurpub/ckab023)
Supplement: ckab023_Supplementary_Data [file ckab023_supplementary_data.zip › ejph-2020-03-om-0274-File005.docx]

***Supplementary Figure 3. Mean BMI by ethnicity and sex. Weighted means unadjusted, 95% CI. N=10500***
