# Supplementary material for: Mid-adolescent ethnic variations in overweight prevalence in the UK Millennium Cohort Study
Source: Eur J Public Health. 2021 Apr 24;31(2):396–402. doi: 10.1093/eurpub/ckab023 (PMC8565477; doi:10.1093/eurpub/ckab023)
Supplement: ckab023_Supplementary_Data [file ckab023_supplementary_data.zip › ejph-2020-03-om-0274-File006.docx]

**Supplementary Table 1.** Comparison of characteristics of full analysis sample versus adolescents with missing data. Proportions unweighted

|  | CM with data missing for at least one variable  **N=1226** | CM with data available for all variables  **N=10500** | *p-value* |
| --- | --- | --- | --- |
|  | Mean (S.E.) | Mean (S.E.) |  |
| **Body Mass Index** | 21.66 *(0.21)* | 21.45 *(0.04)* | *0.2716** |
| **Fat Mass Index** | 5.02 *(0.19)* | 5.01 *(0.03)* | *0.9372** |
|  | % | % |  |
| **Overweight** |  |  |  |
| Healthy weight- including underweight | 71.8 | 73.5 |  |
| Overweight- including obese | 28.2 | 26.5 | *0.415^a^* |
| **Ethnic group** |  |  |  |
| White | 73.9 | 80.0 | *<0.0001^a^* |
| Mixed | 6.3 | 4.6 |  |
| Indian | 2.5 | 2.7 |  |
| Pakistani | 8.1 | 4.9 |  |
| Bangladeshi | 2.7 | 2.2 |  |
| Black Caribbean | 2.1 | 1.0 |  |
| Black African | 2.2 | 1.9 |  |
| Other ethnic group | 2.2 | 2.8 |  |
| **Sex** |  |  |  |
| Male | 48.5 | 50.4 | *0.201^a^* |
| Female | 51.6 | 49.6 |  |
| **Age** |  |  |  |
| 13 | 21.6 | 24.4 | *<0.0001^a^* |
| 14 | 75.5 | 74.5 |  |
| 15 | 2.9 | 1.2 |  |
| **Maternal Education** |  |  |  |
| NVQ 4/5 Higher education | 33.1 | 46.0 | *<0.0001^a^* |
| NVQ 3 e.g. A/AS Level | 14.0 | 14.6 |  |
| NVQ 2 e.g. GCSE grade A-C | 26.5 | 22.6 |  |
| NVQ 1 e.g. GCSE below grade C | 7.2 | 5.5 |  |
| Overseas qualification only | 3.7 | 3.0 |  |
| None of these | 15.6 | 8.3 |  |
| **OECD UK Equivalised Income Quintile** |  |  |  |
| Highest | 12.9 | 23.8 | *<0.0001^a^* |
| Fourth | 16.5 | 23.7 |  |
| Third | 19.2 | 20.5 |  |
| Second | 23.1 | 16.2 |  |
| Lowest | 28.3 | 15.9 |  |
|  |  |  |  |
| ^CM: Cohort member^  ^a^p-values obtained through chi square tests  *p-value obtained through t test | | | |

|  | CM with data missing for at least one variable  **N=1226** | CM with data available for all variables  **N=10500** | *p-value* |
| --- | --- | --- | --- |
| **Birthweight** |  |  |  |
| Low (<2.5kg) | 8.4 | 6.8 | *0.102^a^* |
| Normal( ≥2.5-4kg) | 80.3 | 82.4 |  |
| High(>4kg) | 11.3 | 10.9 |  |
| **Breakfast consumption** |  |  |  |
| Never | 11.4 | 8.4 | *<0.0001^a^* |
| Some days but not all days | 44.5 | 37.2 |  |
| Every day | 44.1 | 54.4 |  |
| **Fruit consumption – at least 2 portions** |  |  |  |
| Never | 9.3 | 8.6 | *0.069^a^* |
| Some days but not all days | 63.5 | 60.3 |  |
| Every day | 27.2 | 31.0 |  |
| **Vegetable consumption- at least 2 portions** |  |  |  |
| Never | 11.4 | 7.7 | *<0.0001^a^* |
| Some days but not all days | 58.9 | 54.3 |  |
| Every day | 29.7 | 37.9 |  |
| **Sugar sweetened beverage consumption** |  |  |  |
| Once a day or more | 25.4 | 23.6 | *0.191^a^* |
| 3-6 days a week | 20.0 | 20.1 |  |
| 1-2 days a week | 23.8 | 24.7 |  |
| Less often but at least once a month | 11.5 | 14.0 |  |
| Less than once a month, hardly ever or never | 19.3 | 17.5 |  |
| **Fast food consumption** |  |  |  |
| Once a day or more | 3.3 | 1.8 | *0.046^a^* |
| 3-6 days a week | 4.7 | 4.4 |  |
| 1-2 days a week | 21.7 | 22.5 |  |
| Less often but at least once a month | 43.2 | 43.9 |  |
| Less than once a month | 21.9 | 23.1 |  |
| Hardly ever or never | 5.2 | 4.3 |  |
| **Physical Activity** |  |  |  |
| 5 days or more | 33.3 | 38.2 | *<0.0001^a^* |
| 3-4 days | 29.9 | 34.0 |  |
| 1-2 days | 29.4 | 23.7 |  |
| Not at all | 7.4 | 4.1 |  |
| ^CM: Cohort member^  ^a^p-values obtained through chi square tests  *p-value obtained through t test | | | |

**Supplementary Table 1 continued.**
